# Randomizing a clinical trial in neuro-degenerative disease

**DOI:** 10.1016/j.conctc.2023.101140

**Published:** 2023-04-20

**Authors:** Anthony C. Atkinson, Belmiro P.M. Duarte, David J. Pedrosa, Marlena van Munster

**Affiliations:** a Department of Statistics, London School of Economics, London WC2A 2AE, United Kingdom; b Polytechnic Institute of Coimbra, ISEC, Department of Chemical & Biological Engineering, Rua Pedro Nunes, 3030–199 Coimbra, Portugal; c Univ Coimbra, CIEPQPF, Department of Chemical Engineering, Rua Sílvio Lima — Pólo II, 3030–790 Coimbra, Portugal; d Department of Neurology, University Hospital Marburg, 35043 Marburg, Germany; e Center of Brain, Mind and Behaviour, Philipps-University Marburg, 35043 Marburg, Germany

**Keywords:** Bias, Biased-coin design, Empirical multivariate distribution, Loss, Minimization, Randomization

## Abstract

The paper studies randomization rules for a sequential two-treatment, two-site clinical trial in Parkinson’s disease. An important feature is that we have values of responses and five potential prognostic factors from a sample of 144 patients similar to those to be enrolled in the trial. Analysis of this sample provides a model for trial analysis. The comparison of allocation rules is made by simulation yielding measures of loss due to imbalance and of potential bias. A major novelty of the paper is the use of this sample, via a two-stage algorithm, to provide an empirical distribution of covariates for the simulation; sampling of a correlated multivariate normal distribution is followed by transformation to variables following the empirical marginal distributions. Six allocation rules are evaluated. The paper concludes with some comments on general aspects of the evaluation of such rules and provides a recommendation for two allocation rules, one for each site, depending on the target number of patients to be enrolled.

## Highlights

• A sample provides the distribution of the (placebo) response and prognostic factors.
• Analysis of the sample data gives a model for analysis of the clinical trial.
• Simulations using the model compare rules for randomized treatment allocation.
• Different randomization rules are proposed for two sites with 184 and 108 patients.

## 1 Introduction

We study methods for randomized treatment allocation for a clinical trial on neuro-degenerative diseases. Two of the best known of such are Alzheimer’s and Parkinson’s diseases. We describe the background to the clinical trial and the forthcoming economic burden of these diseases on advanced societies in the next section. The purpose of this paper is to compare various randomization methods in sequential trials in which patients present with prognostic factors which may be included in the analysis of the data and so should be allowed for in the randomization scheme. We use data from a sample of patients similar to those to be included in the trial

Our focused objective is to provide a scientific basis for the randomization scheme for this particular trial, based on empirical evidence. It is intended that our results will contribute to justify this particular aspect of the trial protocol.

Because we have a clear objective we do not provide a general survey of randomization methods in clinical trials. Such a survey can be found in Rosenberger and Lachin [1]. Several of the methods we compare are derived from forms of randomized treatment allocation introduced by Atkinson [2] using the methods of optimum experimental design. These were extended by Atkinson [3] to include comparisons of the statistical properties of the designs, particularly the loss of efficiency due to randomization and potential bias from the ability to guess the next treatment to be allocated. Both that paper and Rosenberger and Sverdlov [4] contain background material on randomization in sequential clinical trials in the presence of covariates. A recent review of inference after covariate-adaptive randomization is Ma et al. [5]. The review of Sverdlov et al. [6] focuses on the use of the methods of optimum experimental design in clinical trials.

The paper is organized as follows. The medical background and the structure of the proposed two-treatment trial, to be performed at two sites, are described in Section 2, followed in Section 3 by the statistical analysis of the sample values of the five covariates (prognostic factors) which may be used in the analysis of the trial results. The analysis of the sample results shows that two variables are important and that a linear regression model should be appropriate for analysis of the clinical trial. The use of randomized forms of the sequential construction of optimum experimental design in sequential clinical trials is introduced in Section 4. The two important measures of the performance of a trial design, loss and bias, are formalized in Section 4.2. Protection against the biases that can result from the absence of proper randomization is especially important in an unblinded trial such as the one we describe. Section 4.3 presents six allocation rules, ranging from deterministic allocation which minimizes the variance of the estimated treatment difference, to random selection of the treatment to be allocated. We also investigate a randomized version of the minimization rule of Pocock and Simon [7].

We use simulation to compare these six rules. A major novelty of our approach is the use of the empirical sample of potential covariates to provide a sampling distribution of covariates, which have some correlations, rather than assuming independent normal distributions for covariate values. The algorithm is in two stages described in Section 5: sampling of correlated multivariate normal variates is followed by marginal transformation of the sampled normal variates to samples from the empirical marginal distributions of the covariates. The main numerical results on the comparison of allocation rules are in Section 6. Extensions in Section 7 explore (i) the effects of designing for either more or fewer covariates than are used in the analysis; and (ii) how comparisons of trial designs change if independent normal covariates are sampled instead of those with the empirical distribution. Shortcomings of the use of categorized covariates in the analysis of the results from trials are also briefly mentioned. The final section discusses a few more general topics, including a more flexible sampling rule and the use of a concept of admissibility in the comparison of trial designs. An alternative to admissibility is a rule due to Ryeznik and Sverdlov [8]. The paper concludes with recommendations for randomization rules at the two trial sites which have different target numbers of patients.

## 2 Background

We first describe the trial and then, in Section 2.2, introduce the main variables in the statistical analysis of Section 3.

### 2.1 The trial

Part of this project is integrated within a larger study to test the effectiveness of complex interventions. Parkinson’s disease (PD) is one of the most common neurodegenerative diseases, leading to significant disability in patients with motor and non-motor symptoms [9]. PD impacts patients’ health-related quality of life and causes a high burden for (informal) caregivers [9]. It is assumed that there are around 400.000 PD patients in Germany [10], many of whom are older than 65 years. In the future, an increasing number of people suffering from PD in Germany is to be expected [10], [11], while at the same time the life expectancy of patients is also increasing [10]. This fundamental societal change requires the development of new and innovative care strategies for people suffering from PD [11], [12].

In Germany, care coordination is primarily the responsibility of resident neurologists and, in some cases, general practitioners. Commonly, outpatient care is only provided once a quarter by the resident neurologist. The coordination of therapies that are tailored to individual needs and the involvement of specially trained care professionals such as Parkinson nurses are rarely implemented [13]. In the case of non-mobile patients, these deficits are further aggravated as trips to the doctor’s office become ever more challenging as the degree of illness increases; home visits are rare in Germany [14].

However, this situation is not sustainable. Therefore, modern therapies, especially for neuro-degenerative diseases, are increasingly moving towards a holistic approach to patient care [15]. Advances in digital technologies open up new possibilities in the field of healthcare provision and professional collaboration. The attractiveness of digital technologies lies in their ability to mitigate both mobility-related barriers and economic obstacles. Digital solutions are also suitable for evaluating the disease activity of movement disorders, since tests developed for this purpose can easily be implemented within the framework of e-Health solutions. In Germany, there are regionally implemented digital solutions for PD patients, but there are no nation-wide healthcare models [14], [16]. As part of the “ParkProReakt” project, a cross-sectoral, proactive, needs-oriented and technology-supported care model is being developed. The heart of the project will be a digitally supported care model in which a multidisciplinary care team (neurologists, Parkinson nurses, outpatient care service and study nurses) are linked to the patients virtually and in real-life. The aim of this project is to improve healthcare and achieve a measurably improved quality of life for PD patients. In addition, the burden on care givers should be reduced, since the use of digital solutions provides support in assessing changes in the course of the disease. This project is funded under a program of the Federal German government, through the Ministry of Health [17].

The healthcare model is being evaluated as part of a clinical study where we will look at the perceived practicability of healthcare professionals working in the model, the impact on the everyday life of people with PD and the economic benefits as well as the effects on patients quality of life. We will include sequentially a certain number of people at two centres of different sizes. Both centres will include and take care of a number of patients who are divided 1:1 into controls (receiving only standard care) and an intervention group (with the complex care we have developed). Our sample size calculation, in terms of quality of life and according to previous publications (see Kleinholdermann et al. [18], Kleinholdermann et al. [19], Mestre et al. [20], Butterfield et al. [21] among others), is based on a total of 292 people distributed as follows:

• 184 patients in Center 1 (92 receiving standard care and 92 treated in the complex care program);
• 108 patients in Center 2 (54 receiving standard care and 54 treated in the complex care program).

### 2.2 Response and prognostic factors

To provide guidance on the design of the study we collected data on a sample of 144 PD patients representative of those being followed at the Department of Neurology, University Hospital Marburg. These are not the patients who will be enrolled in the trial.

The response variable of the data sample is Quality of life, (QoL), which can be practically measured with two similar questionnaires: (i) the Parkinson’s Disease Questionnaire (PDQ39) with 39 items; and (ii) a PDQ with 8 items. The 8-item disease questionnaire, with response *pdq8*, [22] is the patient reported outcome measure constructed by taking one question from each of the eight domains of PDQ39 [23]. Of course both are an oversimplification being a reduction of the abstract QoL. Both metrics have been extensively used [24], [25]; PDQ39 allows a better and wider characterization of QoL than PDQ8, but the latter is practically easier to measure. Unfortunately, we do not routinely administer the PDQ39 questionnaire, so we cannot explore the loss of information in using the shorter version (PDQ8). Several authors report a strong correlation between the results of the two, for instance Chen et al. [26]. In our trial the primary endpoint is *pdq8* which measures the QoL on a percentage scale (0-100 %), higher values showing reduced quality of life.

Now, we introduce the prognostic factors. The literature recognizes various important factors related with: (i) disease duration and the stage of disease; (ii) psychological well-being/neuropsychiatric symptoms (depression, anxiety); (iii) demographic metrics (age, gender, area of living, income) and (iv) cognitive impairment. The first, here denoted by *h&q*, is measured on the Hoehn and Yahr scale, see [27], [28]. Increasing values indicate more severe affection on an ordinal scale. The second, *bdi*, measures the symptoms of depression via Beck’s Depression Inventory (BDI). This indicator is determined from a questionnaire, see Beck et al. [29]. Values from 10 and higher indicate increasing levels of depression. Although many different aspects might be expected to influence quality of life, recent literature has highlighted these two factors as important determinants [13], so a balance between them may be indicated. In this paper, we extend this list and include three more variables in the data analysis of the next section, namely *age*, *gender* and *moca*, that is cognitive impairment measured by the Montreal Cognitive Assessment (MoCA) test [30]. Resources were not available to measure further variables.

**Fig. 1:**
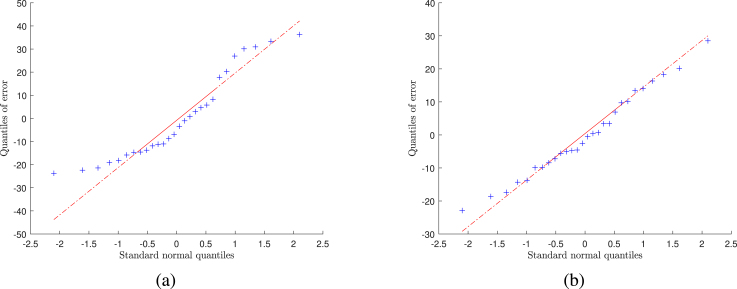
Normal probability plots of residuals from two fitted models: (a)*pdq8* vs. *bdi*; (b)*pdq8* vs. *h&y* and *bdi*.

## 3 Data modelling

Because our allocation rules depend on a statistical model of the data, we start with an analysis of the data on the response and the five potential prognostic factors. The analysis leads to building a regression model.

There are data from 144 patients on the response *pdq8* and on the five prognostic factors. The correlations between all variables are shown in Table 1. The most noticeable features are the correlations of 0.640 and 0.332 between the response *pdq8* and two of the prognostic factors, *bdi* and *h&y*. These two variables, in turn, have a correlation of 0.325.

The regression of *pdq8* on all five prognostic factors produces the results reported in Table 2. The order of the appearance of the covariates in Table 2 is that of their inclusion in the linear model obtained via stepwise regression. Surprisingly, in the light of the correlations in Table 1, there is significant regression on *bdi* but not on *h&y*; due to the correlation between the covariates, much of the variability of *pdq8* explained by *bdi* is already explained by *h&y*.

**Table 1:** Correlation matrix between the response and prognostic factors.

	*gender*	*age*	*h&y*	*bdi*	*moca*	*pdq8*
*gender*	1.0000	0.0792	0.0180	−0.1218	−0.0376	−0.0807
*age*		1.0000	0.2266	−0.0513	−0.4766	−0.1415
*h&y*			1.0000	0.3250	−0.6435	0.3318
*bdi*				1.0000	−0.2689	0.6402
*moca*					1.0000	0.0419
*pdq8*						1.0000

We checked several models using normal probability plots of the residuals. The left-hand panel of Fig. 1 shows the normal quantile–quantile (QQ) plot of the residuals from least squares regression on just *bdi* and the right-hand panel shows a similar plot from regression on both *bdi* and *h&y*. The plot from regression on two variables is appreciably straighter, indicating a more nearly normal distribution of residuals. This plot is also straighter than that of the residuals from regression on all five variables (not shown). In our exploration of methods for balancing and randomizing treatment allocations we therefore take as our standard allocations those using just two prognostic factors with homoscedastic independent normal errors. However, in Section 7.1 we also briefly consider the effect of allocations using fewer or more prognostic factors.

**Table 2:** Order of addition of covariates to *pdq8* model (via stepwise regression).

Order of addition	Source	SSE	SSR	d.f.	MSE	MSR	F	Prob>F
1	Intercept			1				
2	*bdi*	4.4768 × 10^3^	5.5662 × 10^3^	1	172.1852	5.5662 × 10^3^	32.3266	5.560 × 10^-6^
3	*age*	4.4280 × 10^3^	5.6150 × 10^3^	1	177.1190	48.8340	0.2757	0.6041
4	*gender*	4.3909 × 10^3^	5.6521 × 10^3^	1	182.9536	37.0898	0.2027	0.6566
5	*h&y*	4.3756 × 10^3^	5.6674 × 10^3^	1	190.2426	15.3065	0.0805	0.7792
6	*moca*	4.3593 × 10^3^	5.6836 × 10^3^	1	198.1522	16.2311	0.0819	0.7774

SSE — sum of square error; SSR — sum of squares of treatments; d.f. — degrees of freedom; MSE — mean square error (MSE=SSE/(n-d.f.));

MSR — incremental mean of squares of treatments (MSR=SSR${}_{i}$-SSR${}_{i-1}$); F — F ratio (F=MSR/MSE).

Although there are partial data on 144 patients, the QQ plots in Fig. 1 present 28 points from regressing pdq8 on one and two regressors. To produce the plots we used only those patients with complete records, that is patients with known h&y and bdi.

**Fig. 2:**
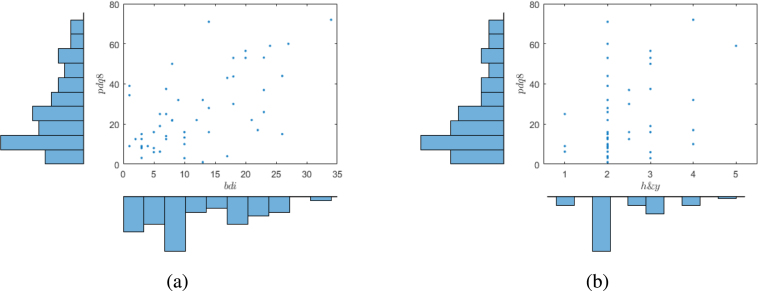
Scatterplots with histograms for: (a)*pdq8* vs. *bdi*; (b)*pdq8* vs. *h&y*.

As a final introduction to the structure of the data used in our simulations of designs, we give in Fig. 2 scatterplots of the response *pdq8* against *bdi* and *h&y*, together with histograms of the distributions of the variables. As is to be expected from the analyses given above, the stronger relationship is between *pdq8* and *bdi*. Note that there are different numbers of points in the two plots. The marginal distribution of *pdq8* is not normal. Normality is revealed by the residuals from joint regression on these two prognostic factors.

The plots in Fig. 2 also reveal that the distributions of *bdi* and *h&y* are not particularly normal. This comment is important when, in Section 7.2, we explore the properties of designs using normally distributed covariates. Similar plots for *age* and *moca* show no relationship with the response *pdq8*.

## 4 Experimental design

### 4.1 Sequential optimum experimental design

Patients arrive sequentially. Patient i presents with a vector of q−1 prognostic factors $z_{i}$ and is allocated to one of two treatments, $\tau_{1}$ or $\tau_{2}$; the response (here, *pdq8*) for this patient is $y_{i}$. The parameter of interest is the treatment difference $\Delta =\left(\tau_{1}-\tau_{2}\right)/2$. The regression model for all n observations, in matrix form, is

(1) $$E\left(Y\right)=a\Delta +I\beta_{0}+Z\psi =a\Delta +F\beta =G\;\omega .$$

In this model a is the n×1 vector of allocations with elements +1 and −1, depending on whether treatment 1 or treatment 2 is allocated, and I is the n×1 vector of ones. The average effect of the two treatments, written as the constant term $\beta_{0}=\left(\tau_{1}+\tau_{2}\right)/2$, is not of importance. The parameter vector ψ of regression parameters for the prognostic factors is also unimportant, although some balance is required over these variables, which will be included in the analysis of the data. The constant and covariates are included in the n×q matrix F. The value of q is important in determining the properties of some allocation rules.

In sequential treatment allocation the covariates and allocations are known for the first n patients, giving a matrix $G_{n}$ of allocations and explanatory variables in (1). Let patient n+1 have a vector $z_{n+1}$ of explanatory variables. If treatment j is allocated, the vector of allocation and explanatory variables for the (n+1)st patient is $g_{j,n+1},\;j=1,2$. Results in the sequential construction of optimum experimental designs (see Atkinson [2] and Smith [31, Section10]) show that the variance of the estimate $\overset{ˆ}{\Delta }$ after n+1 observations is minimized by the choice of that treatment for which the sensitivity function

(2) $$d_{s}\left(j,n,z_{n+1}\right)=g_{j,n+1}^{T}{\left(G_{n}^{T}G_{n}\right)}^{-1}g_{j,n+1}-f_{j,n+1}^{T}{\left(F_{n}^{T}F_{n}\right)}^{-1}f_{j,n+1}$$

is a maximum. This result is a special case of the use of optimum design theory to minimize the variance of a single parameter estimate in a model with several nuisance parameters, a criterion called D${}_{\text{s}}$-optimality. See Atkinson et al. [32, §10.3] with s=1.

Once the prognostic factors are known for patient n+1, treatment allocation in the sequential optimum design of experiments is determined. This procedure leads to a trial in which the variance of $\overset{ˆ}{\Delta }$ is minimized; there is no allowance for randomization. Randomness in the allocations will provide protection against biases and unexpected trends, but at the cost of a slight loss in efficiency, that is an increase of the variance of $\overset{ˆ}{\Delta }$.

### 4.2 Assessing rules: Bias and loss

The loss from randomization is assessed from Var($\overset{ˆ}{\Delta }$). Let $b=F^{T}a$, a “balance” vector which is identically zero when all covariates are balanced across all treatments, which is a consequence of the sequential construction of Section 4.1 for the linear model (1). Then

(3) $$\text{var}\left(\overset{ˆ}{\Delta }\right)=\frac{\sigma^{2}}{n-b^{T}{\left(F^{T}F\right)}^{-1}b}=\frac{\sigma^{2}}{n-L_{n}},$$

giving an explicit expression for calculation of the loss $L_{n}$. The loss is minimized for the balanced design when the estimate of Δ is independent of the estimates of the nuisance parameters. As (3) indicates, the loss quantifies the number of patients on whom information is effectively lost due to imbalance in the trial.

The loss $L_{n}$ in a specific trial depends on the particular sequence of randomized allocations. In this paper, interest is in comparing the properties of various allocation rules, so that the focus is on the expectation $E\left(L_{n}\right)=L_{n}$, approximated by ${\overset{¯}{L}}_{n}$, the average over $n_{\text{sim}}$ simulations. For some allocation rules theory provides a value for the expected value of the loss $L_{n}$ as n→∞. However, even in such cases, simulation is informative about trials for moderate values of n.

A numerical measure for randomization is selection bias [33] which measures the ability to guess the next treatment to be allocated. Bias depends on the design, the guessing strategy and, for some rules, the value of n. For a particular combination of strategy and design the expected bias $B_{n}$ is estimated from $n_{\text{sim}}$ simulations as

(4) $${\overset{¯}{B}}_{n}=\frac{\text{number}\;\text{of}\;\text{correct}\;\text{guesses}\;\text{of}\;\text{allocation}\;\text{to}\;\text{patient}-\text{number}\;\text{of}\;\text{incorrect}\;\text{guesses}}{n_{\text{sim}}}.$$

This definition is similar to that of (4.2) of Smith [31]. The guessing strategy used in our numerical comparisons is the sensible one of guessing that the treatment for which the allocation probability is higher will be selected.

Amongst many others, Efron [34] and Smith [31] consider that selection bias should not be an issue in double-blind trials with treatment allocation made remotely from the trial, although it may be if there are local attempts towards institutional balance [35]. It is however impossible to blind the trial with which we are concerned. Allocation may be blinded, but the patient and medical staff will know without doubt which treatment has been allocated. For us, then, randomization is particularly important. In general, a trial without randomization appears to lack objectivity. Efron [34] and Smith [31] accordingly study the effect of biased-coin designs on freedom from accidental bias due to omitted factors including time trends and, in the case of Smith [31], correlated errors and outliers. The conclusion of Smith [31] is that biased-coin designs that are not completely random provide good protection against several sources of bias and that selection bias is a good measure of the properties of the design.

Randomization and balance are in conflict. The deterministic rule of sequential optimum design minimizes loss. However, the allocation can always be correctly guessed, so that $B_{n}=1$. The antithesis is the random rule in which the treatment is allocated by the toss of a fair coin. This has the maximum loss of all rules we consider, but it is impossible to have any systematic success in guessing the next allocation, so that $B_{n}=0$. In this paper we study several design strategies intermediate in properties between sequential optimum design and random allocation.

### 4.3 Six allocation rules

We now describe the six rules that we compare in a variety of scenarios for randomizing the experiment. Some of the rules are based on the sequential construction of the optimum design for estimation of Δ. Let the treatment maximizing (2) be $\tau_{\text{[1]}}$, which is allocated with probability π([1]).

*Rule D: Deterministic Allocation.* This is the sequential construction of the D${}_{\text{s}}$-optimum design; $\pi_{\text{D}}\left(\left[1\right]\right)=1$. It follows that $L_{\infty }=0$ and, since there is no randomization, $B_{\infty }=1$. The simulations in later sections show that, from very small values of n, ${\overset{¯}{L}}_{n}≅0$ and ${\overset{¯}{B}}_{n}≅1$.

*Rule A: Randomized D*${}_{\text{A}}$*-optimality.* Atkinson [2] introduced a randomized form of the sequential construction of D${}_{\text{A}}$-optimum designs. For two treatments the probability of allocation of treatment j is

(5) $$\pi_{\text{A}}\left(j\right)=\frac{d\left(j,n,z_{n+1}\right)}{d\left(1,n,z_{n+1}\right)+d\left(2,n,z_{n+1}\right)}.$$

Burman [36] showed that for this rule $L_{\infty }=q/5$. The values of $d\left(1,n,z_{n+1}\right)$ and $d\left(2,n,z_{n+1}\right)$ are not standardized by n. As n increases the difference between the two decreases and as $n\to \infty ,\pi_{\text{A}}\left(j\right)\to 0.5$. As a consequence, $B_{\infty }=0$.

*Rule E: Efron’s Biased Coin.* Efron [34] introduced a design for the sequential comparison of two treatments , without covariates, in which the under-represented treatment was allocated with probability 2/3. In the presence of covariates let the under-represented treatment be denoted [1]. Then

(6) $$\pi_{\text{E}}\left(\left[1\right]\right)=2/3$$

The loss decreases with n but, from small n, the values of $B_{n}$ are close to the asymptotic value of 1/3.

*Rule MwC: Minimization with a Coin.* The deterministic minimization rule of Pocock and Simon [7] depends on calculating the total effect on all measures of marginal imbalance when treatment j is allocated. With q−1 covariates z, there will be q−1 measures to be summed. The individual measures count the number of observations in each category of the covariate. Continuous covariates therefore have to be categorized.

Let the total effect on imbalance be C(j). The allocations are ranked so that C([1])≤C([2]). In this deterministic allocation treatment [1] is allocated, with random allocation if both treatments have the same value of C(j).

We introduce randomization by replacing certain allocation by the 2/3 of Efron’s biased coin. Thus

(7) $$\pi_{\text{MwC}}\left(\left[1\right]\right)=2/3,$$

with random allocation if there is a tie, as there may well be, since the prognostic factors are discretized. The deterministic calculations are exemplified by [3], [37] as well as by [7].

*Rule R: Randomized Allocation.*$\pi_{\text{R}}\left(\left[1\right]\right)=0.5$. This is the furthest in properties from deterministic allocation, Rule D. Now since there is complete randomization, $B_{\infty }=0$. A special case of the calculations in Burman [36] is that $L_{\infty }=q$, a result that goes back at least to Cox [38].

*Rule RwS: Randomized Allocation within Strata.* This is rule similar to rule R but allocates the individuals in each stratum. That is the most important factors in pdq8 regression are discretized using the median value as cut-off and the allocation of patients based on the complete randomization in each (of the four) strata.

The randomization in Rules MwC and RwS depends upon stratification of the covariates. In the comparisons of Section 6.2, it is assumed that the unstratified variables are used to fit the data. At the end of Section 7.2 we briefly consider the effect of using the stratified variables in modelling.

## 5 Sampling from the multivariate empirical distribution of prognostic factors

Simulation is often used, as here, to find the small sample properties of treatment allocation procedures. Many such investigations, such as Atkinson [3], [39], assume that the prognostic factors are uncorrelated and normally distributed. Here we sample from an approximation to the empirical correlated distribution of the prognostic factors analysed in Section 3. In the absence of the empirical distribution the procedure is unchanged, except that sampling is from a prior distribution, preferably based on some empirical evidence.

In general, it is difficult to sample from multivariate distributions with arbitrary covariances. One possibility is to sample, with replacement, from the q−1 dimensional discrete distribution of the observed covariates. An alternative, which gives more sampling points, is to generate a q−1 dimensional multivariate normal sample with the desired correlation and then to transform the normal distributions to have the univariate empirical distributions discussed in Section 3.

Let the q−1 prognostic factors of patients entering the trial have the correlation matrix, Γ, extracted from the data in Table 1. Let u be a q−1 vector of uncorrelated standard normal variables. To generate a vector of correlated normal random variables v, we first decompose Γ using the Cholesky decomposition, i.e. $\Gamma =\Lambda \;\Lambda^{T}$, where Λ is a (q−1)×(q−1) lower triangular matrix. We then form the elements of the correlated normal q vector for a new patient using the rule

(8a) $$v_{1}=1.0$$

(8b) $$v_{i}=\overset{q-1}{\underset{j=1}{\sum }}\Lambda_{i-1,j}\;u_{j},\;i=2,\ldots ,q,$$

where (8a) is for the constant term and (8b) is for the prognostic factors.

We now further transform the $v_{i},i=2,\ldots ,q$ to have the desired empirical distribution. Let the ordered vector of sampled values of the empirical prognostic factors of Section 3 for variable i be $s_{i}$. Then $\ldots s_{i,k-1}<s_{i,k}<s_{i,k+1}\ldots$ with cdf $F_{i}\left(s_{i,k}\right)=P\left(S_{i}\leq s_{i,k}\right)$. We sample the distribution of $S_{i}$ using the cdf of the normal distribution of $v_{i}$ to provide the probabilities for our correlated sample. That is, let $p_{i}=\Phi \left(v_{i}\right)$, where Φ is the cdf of the standard normal distribution. Then the values of the simulated covariates $z_{i}$ are found by numerical search:

(9) $$\text{if}\;F_{i}\left(s_{i,k-1}\right)<p_{i}\leq F_{i}\left(s_{i,k}\right),\;z_{i}=s_{i,k},\;i=2,\ldots ,q,$$

with $z_{1}=1.0$.

In our analyses we consider: (i) q=6, that is including all the prognostic factors when samples are generated from a five-variate normal distribution; (ii) q=3, including the variables *h&y* and *bdi* and so sampling from a bivariate normal distribution and (iii) q=2, only the variable *bdi* is used for prediction of *pdq8*. In this case samples come from a standard normal distribution and Γ=[1.0]. For non-correlated prognostic factors we consider only the normal case and put $z_{i}=v_{i}$ in (8) with $\Gamma =I_{q-1}$ where $I_{q-1}$ is the q−1 identity matrix.

## 6 The trial design and comparison of allocation rules: Empirical prognostic factors

### 6.1 The overall design of the sequential trial

There are two sites for the trial. Budget constraints and power considerations led to a design in which one site is expected to enrol 184 patients and the other 108. It is sensible to randomize separately for the two centres. One reason is that of robustness of the procedure. It will be more straightforward to run two separate schemes, rather than to rely on communication between the centres and the transfer of covariate information. Practically, separate randomization schemes reduce the probability of confusion and errors. The second reason is that it is possible the distribution of covariates at the two site may be different — perhaps due, for example, to socio-economic or demographic factors. In the data analysis we need to be prepared to be able to fit models to well-balanced data from the individual sites, as part of the process that, it is to be hoped, will lead to a single model and analysis for all patients.

The properties of randomization rules depend on the number of patients in the trial. Since it is not certain that the two centres will be able to recruit exactly the specified number of patients, we compare the properties of randomization rules for values of n up to 184. These results are given graphically. Because, however, there are two specific target values of n, we also provide tabulations of the properties of the rules for n=108 and 184.

### 6.2 Comparison of allocation rules: Empirical prognostic factors

We start our comparison of the allocation rules taking q=3, that is the intercept and the two prognostic factors *bdi* and h&y which are most highly correlated with the response. There were 20.000 simulations in all comparisons.

**Fig. 3:**
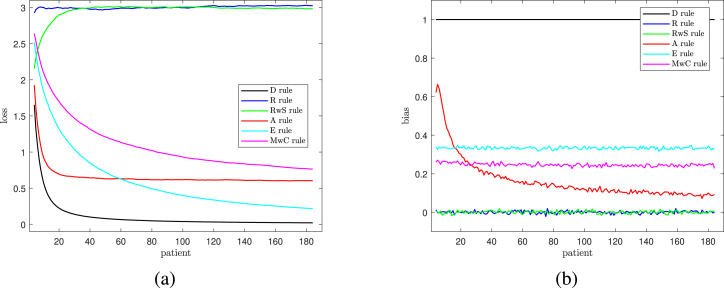
Results for model including 3 parameters (the intercept plus 2 the two covariates to h&y and *bdi* – those with the largest correlation with the response): (a) Loss and (b) Bias, as functions of the number of patients.

The results are plotted in Fig. 3 and summarized in the central panel of Table 3. The left-hand panel of the figure shows the loss for values of n up to 184. Rule R has a loss of 3 (=q) throughout, in line with the results quoted in Section 4.3; the loss for RwS rapidly increases to be indistinguishable from that for R. Reading down in the centre of the plot, the loss for Rule MwC is gradually decreasing, being slightly less than one at n=184. The loss for Rule A settles to a value of q/5=0.6 just after n=108. The loss for Rule E decreases steadily, becoming less than that for Rule A when n is close to 60. It is however always greater than that for Rule D, for which $L_{\infty }=0$.

The right-hand panel of the figure shows the plot for bias. This has a simpler structure. Rules R, RwS, E and D have constant biases of 0, 0, 1/3 and 1, within sampling fluctuation. The bias for MwC is also constant, but lower than that for Rule E because of the occurrence of ties; the value is close to 0.25 rather than 1/3. The bias for Rule A, unlike the others, decreases steadily, in line with the argument of Section 4.3. The close similarity of R and RwS is a feature of all comparisons; we return to this point at the end of the section.

**Fig. 4:**
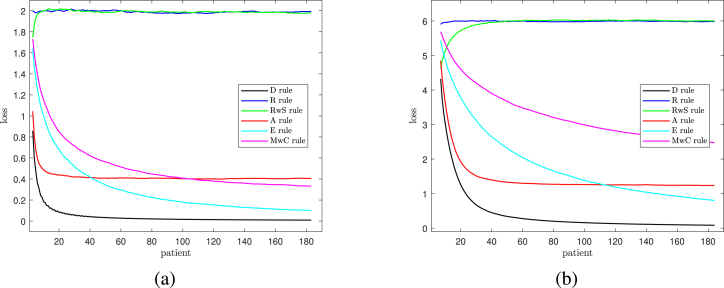
Losses as a function of n. Left-hand panel q=2 (just *bdi*) and Right-hand panel q=6 (all prognostic factors).

We also investigated the properties of the six rules for two further values of q. The two panels of Fig. 4 show the plots of loss for q=2 and q=6. Now the losses for Rule R are two and six and those for Rule A tend to 0.4 and 6/5 for large n. For q=2 (the left-hand panel) the losses all proportionately decrease faster than they do for the right-hand panel. This effect is particularly marked for the two Rules MwC and E that randomize using Efron’s coin. The biases for both values of q are similar in structure to those for q=3 in the right-hand panel of Fig. 3 and so are not shown here.

**Table 3:** Performance of allocation rules after 108 and 184 patients (model with correlated empirical covariates).

Covariates	Rule	After 108 patients		After 184 patients	
		Loss	Bias	Loss	Bias
1	D	0.0149	1.0000	0.0086	1.0000
	R	1.9837	−0.0041	1.9979	0.0009
	RwS	1.9809	−0.0016	1.9838	0.0035
	A	0.4011	0.1132	0.4072	0.0751
	E	0.1706	0.3330	0.1036	0.3288
	MwC	0.4967	0.2598	0.4421	0.2448

2	D	0.0360	1.0000	0.0209	1.0000
	R	3.0047	−0.0012	3.0300	−0.0001
	RwS	3.0301	−0.0098	3.0243	0.0040
	A	0.6157	0.1157	0.6042	0.0941
	E	0.3673	0.3336	0.2202	0.3280
	MwC	1.1030	0.2419	0.9768	0.2407

5	D	0.1483	1.0000	0.0848	1.0000
	R	5.9836	−0.0106	5.9980	−0.0035
	RwS	6.0220	−0.0002	5.9743	−0.0134
	A	1.2633	0.1728	1.2167	0.1397
	E	1.3253	0.3332	0.8210	0.3352
	MwC	3.0433	0.2990	2.6117	0.3004

Values of both loss and bias for q=6,3 and 1 and n=108 and 184 are in Table 3, these being the two values of importance for the trial on neuro-degenerative diseases. The table confirms the suggestion of the figures that Rule A provides a good compromise between loss and bias, low values of both of which are desirable. More generally, the losses for Rules E and MwC in the right-hand panel of Fig. 4 show the poor performance of these two rules as q increases.

Finally, we consider the close relationship between Rules R and RwS. In the latter, the covariates are categorized into $2^{q-1}$ cells. Any particular patient will have a treatment randomly assigned within the appropriate cell; cell membership is then ignored in the analysis of the data. Consequently, there is no effect of the cell and no difference between Rules R and RwS.

## 7 Extensions

### 7.1 Design for an incorrect number of prognostic factors

It may be that a trial is designed with randomization over q prognostic factors but the final data analysis incorporates r factors, where r may be greater than, or less than q. Results for allocations using the six rules are in Table 4. The top half of the table is when extra covariates are included in the design: balancing is over five covariates, but only two are used in the data analysis. Rule R does not depend on the value of q and here, with r=3, the loss of this rule is very close to three. The losses for the other rules are close to two. There is little effect of n, but the loss for Rule D is lowest. The next highest losses after R are 1.1163 for MwC when n=108 and 0.9245 when n=184. The lower half of the table is for the reverse situation, when two are included in the design, but the analysis has r=6. Now the losses for Rule R are just above six, with the other rules giving losses round about five. Again Rule D gives the lowest losses with Rule MwC now giving the highest values, 3.9348 and 3.7728 for the two values of n and Rule A not too far. The conclusion is that, in order to obtain the benefit of a rule balancing bias and loss, it is important to design for the variables that will eventually be used in the analysis.

There is some recent theoretical work on the properties of designs when r>q, that is “what is the effect of the randomization on the non-randomized covariates?”. Unfortunately, this work does not cover our situation as Liu and Hu [40] only consider discretized covariates and Ye et al. [41] develop a model-free approach. Both papers usefully present details of recent work on covariate-adaptive randomization.

**Table 4:** Performance of allocation rules after 108 and 184 patients when the design is obtained with q covariates and used in a model including r covariates (models with correlated empirical covariates).

Covariates (q/r)	Rule	After 108 patients	After 184 patients
		Loss	Loss
6/3	D	0.0703	0.0402
	R	2.9709	2.9877
	RwS	3.0016	3.0043
	A	0.6221	0.6169
	E	0.6284	0.3850
	MwC	1.1163	0.9245

3/6	D	3.1025	3.0591
	R	5.9956	6.0308
	RwS	6.0072	5.9794
	A	3.6977	3.6397
	E	3.4379	3.2565
	MwC	3.9348	3.7727

### 7.2 Independent normal covariates

Many simulation studies of treatment allocation in clinical trials, such as Atkinson [39] have taken the prognostic factors to be independently normally distributed. We now check whether, in our example, the more complicated simulation strategy we have used leads to results distinct from those from the simple assumption of normality.

The results for simulations with independent normal prognostic factors when q=3 are in Table 5. Comparison with the central panel of Table 3 shows only a few slight differences between the use of independent normal prognostic factors and the correlated empirical factors coming from the data. The two largest differences are in the reduction in loss for Rule MwC when normal covariates are used.

**Table 5:** Performance of allocation rules after 108 and 184 patients (model with two non-correlated normal covariates.

Covariates	Rule	After 108 patients		After 184 patients	
		Loss	Bias	Loss	Bias
2	D	0.0355	1.0000	0.0207	1.0000
	R	3.0015	−0.0012	3.0274	−0.0001
	RwS	3.0127	−0.0098	2.9886	0.0040
	A	0.6145	0.1081	0.6012	0.0896
	E	0.3670	0.3336	0.2197	0.3280
	MwC	0.8907	0.2442	0.7388	0.2372

It is a matter for further exploration as to how general is this result. For methods that allocate according to a function of the information matrix of the design, it is clear that the distribution of the factors will have little effect on the value of loss as n→∞, provided the distribution of the covariates has a finite variance. The behaviour of minimization, without randomization, which we did not consider, depends strongly on the distribution and correlation structure of the prognostic factors. Some details are in Figure 2 and Table 2 of Atkinson [3]. However, minimization is not sensitive to a binary covariate, in our case *gender*. These results also demonstrate the lack of sensitivity of values of loss from Rules R, A and D to the marginal distributions and correlation of the prognostic factors.

The results in Atkinson [3] assume that, however the randomization is achieved, the model is fitted with uncategorized covariates. Categorization of the covariates for fitting is not in general to be encouraged [42]. Even if a symmetrically distributed covariate is categorized about its true median, there is an appreciable loss in information. This is a loss for each observation, so that $L_{n}\to \infty$ with n. The efficiency is further reduced if the distribution is skew when the important information that comes from the tails of the distribution is ignored in fitting the model with categorical variables. Furthermore, the evaluation of the categorization points in the light of the data leads to problems with the levels of significance tests.

## 8 Discussion

The purpose of our paper is to compare the performance of several randomization rules for treatment allocation for a specific clinical trial on treatment of neuro-degenerative diseases. We were fortunate in having available a preliminary set of data from which we were able to estimate the empirical distribution of the prognostic factors. In order to simulate from this empirical distribution, as we describe in Section 5, we sampled from correlated normal random variables which were then transformed to have the desired marginal distributions. As far as we know this procedure has not previously been used in the context of randomizing treatment allocation in clinical trials. The results of our simulations suggest the use of randomized forms of sequential design construction based on D- or D${}_{s}$-optimality.

Use of the preliminary set of data provides an analysis of randomization procedures based on the appropriate model for these data. As we mention in Section 1, in the absence of such information, studies of randomization procedures customarily assume a multivariate normal distribution of the prognostic factors. Results in Table 5 indicate, for our example, that a similar assessment of the relative merits of the different rules is obtained with such a distribution. Simulation results in §4.4 of Atkinson [3] show that discrete or skew covariate distributions have a small effect on comparisons of the rules.

There are many other allocation rules that have been studied in the reviews mentioned in Section 1. One possibility is to use a different function of $d_{s}\left(.\right)$ (2) in the definition of the allocation probability. Atkinson [3] developed ideas on the balance between randomness and information in Ball et al. [43] to replace (5) with the Bayesian form

(10) $$\pi_{\text{B}}\left(j\right)=\frac{{\left\{1+d\left(j,n,z_{n+1}\right)\right\}}^{1/\gamma }}{\overset{2}{\underset{k=1}{\sum }}{\left\{1+d\left(k,n,z_{n+1}\right)\right\}}^{1/\gamma }}.$$

An advantage of this rule is that initially, for small n, the allocations force balance at the cost of high bias. As n increases the allocation moves towards low bias and a higher loss, although with a proportionately smaller loss for values standardized by n. This rule is particularly appropriate if it is not known when the trial is likely to stop. The rate of change of emphasis in the allocation depends on the value of the parameter γ. A suitable value for a specified n can be determined by simulation.

In general, all rules involve a trade-off between bias and loss. Comparisons are helped by the use of the normalized loss, scaled to lie between zero and one:

Normalized loss=Loss/q.

**Fig. 5:**
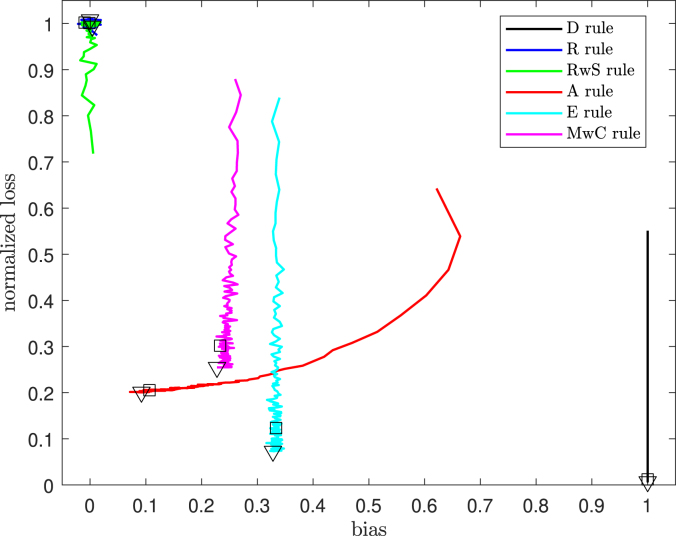
Normalized loss vs. bias for q=3: empirical correlated covariates. The symbol “□” indicates the performance after 108 patients, and “▿” the performance after 184 patients.

Fig. 5 presents the normalized loss vs. bias for all rules for q=3. As we have seen from earlier figures, the comparative properties of the rules depend upon the value of n. We have marked the values for n=108 and 184 on the plot. It is clear that, for all rules except R and RwS, increasing n leads to decreasing loss. It is also clear from the closeness of the plotted symbols for n=108 and n=184 that the majority of the change in properties occurs for small values of n.

The concept of the admissibility of a rule [3] is helpful in interpreting such plots. Small values of both loss and bias are desirable: if Rule 2 has higher levels of bias and loss than Rule 1, then Rule 2 is inadmissible. Rules D and R are always admissible, since they respectively have the minimum values of loss and bias. Fig. 5 shows that Rule MwC is inadmissible compared with Rule A, for both values of n of interest. Rule E has lower loss for these values of n than does Rule A. However, the bias is greater; admissibility does not provide a rationale for preferring one of the designs to the other.

The situation in the trial of this paper is simple; we require to find good allocation rules for just two values of n; 108 and 184. A source of guidance comes from Table 6 which gives results, extracted from Table 3, for Rules A and R, where loss is expressed as a percentage of the number of patients. For Rule A the percentage loss when n=108 is 0.57, rising to 2.78 if Rule R is used. This number may well represent too large a loss of information despite the value of zero for bias. However, when n=184, the percentage loss for Rule R is only 1.64. One might therefore suggest that Rule A be used for the centre with 108 patients and Rule R for the centre with 184 patients, the larger sample size leading to a lower potential bias from allocation. Since the centres are randomizing independently, we see no reason why the two sites should follow the same allocation rule.

**Table 6:** Performance of allocation rules A and R for 108 and 184 patients — percentage of loss per patient.

Rule	For 108 patients		For 184 patients	
	% Loss	Bias	% Loss	Bias
A	0.57	0.11	0.33	0.05
R	2.78	0.00	1.64	0.00

The values of bias and normalized loss both lie between zero and one. Ryeznik and Sverdlov [8, §3.3] suggest a quantification of the distance from the (bias, normalized loss) point to the unachievable origin (0,0), which is the “ideal point” for the two criteria. See also Berger et al. [44]. They suggest a scaled Euclidean distance, which we rescale by √2 to give the measure

(11) $$BL={\left\{{\left(\text{bias}\right)}^{2}+{\text{(normalized loss)}}^{2}\right\}}^{0.5},$$

for which R and D both have the value 1. The results are in Table 7.

For this particular weighting of bias against loss, the results show that Rule A is best for both values of n, as is also evident on inspection of Fig. 5. The procedures of this paper present methods for selecting a randomization rule for the allocation of treatments that can respond to the clinician’s assessment of the relative importance of bias and loss.

**Table 7:** Distances BL (7) from the rules to point (0,0).

Number of patients	Rules					
	D	R	RwS	A	E	MwC
108	1.0001	1.0016	1.0101	0.2356	0.3554	0.4401
184	1.0000	1.0100	1.0081	0.2223	0.3361	0.4049

## Declaration of Competing Interest

The authors declare that they have no known competing financial interests or personal relationships that could have appeared to influence the work reported in this paper.
